# Enteral citrulline supplementation versus placebo on SOFA score on day 7 in mechanically ventilated critically ill patients: the IMMUNOCITRE randomized clinical trial

**DOI:** 10.1186/s13054-023-04651-y

**Published:** 2023-10-03

**Authors:** Jean-Marc Tadié, Clara Locher, Adel Maamar, Jean Reignier, Pierre Asfar, Morgane Commereuc, Mathieu Lesouhaitier, Murielle Gregoire, Estelle Le Pabic, Claude Bendavid, Caroline Moreau, Jean-Luc Diehl, Alain Gey, Eric Tartour, Yves Le Tulzo, Ronan Thibault, Nicolas Terzi, Arnaud Gacouin, Mikael Roussel, Christophe Delclaux, Karin Tarte, Luc Cynober

**Affiliations:** 1 UMR 1236, Univ Rennes, INSERM, Établissement Français du Sang, LabexIGO, Rennes, France; 2 SITI Laboratory, CHU Rennes, Rennes, France; 3 Réanimation Médicale, CHU Rennes, Rennes, France; 4 Centre d’investigation clinique de Rennes (CIC1414), CHU Rennes, Rennes, France; 5 Inserm, Centre d’investigation clinique de Rennes (CIC1414), service de pharmacologie clinique, Institut de recherche en santé, environnement et travail (Irset), UMR S 1085, EHESP, Univ Rennes, CHU Rennes, 35000 Rennes, France; 6 MIP, UR 4334, Médecine Intensive Réanimation, Nantes Université, CHU Nantes, 44000 Nantes, France; 7 Département de Médecine Intensive - Réanimation et Médecine Hyperbare, Centre Hospitalier Universitaire, Angers, France; 8 Service de Réanimation Médicale, Assistance Publique-Hôpitaux de Paris (AP-HP), Hôpital Européen Georges Pompidou, Paris, France; 9 Laboratoire de Biochimie-Métabolique, CHU Rennes, Rennes, France; 10 INSERM U970, Université Paris Cité, Paris, France; 11 Hôpital européen Georges Pompidou, Service d’Immunologie biologique, 20, Rue Leblanc, 75015 Paris, France; 12 INSERM, INRAE, Nutrition Métabolismes et Cancer, NuMeCan, Univ Rennes, CHU Rennes, Service d’endocrinologie-Diabétologie-Nutrition, Rennes, France; 13 AP-HP, Hôpital Robert Debré, Service de Physiologie Pédiatrique -Centre du Sommeil - CRMR Hypoventilations alvéolaires rares, INSERM NeuroDiderot, Université de Paris, 75019 Paris, France; 14 Faculty of Pharmacy, Paris Cité University, Paris, France; 15 Centre Hospitalier Universitaire, Université de Rennes 1, Rennes, France; 16 Hôpital Pontchaillou, CHU Rennes, 2 rue Henri Le Guillloux, 35033 Rennes Cedex, France

**Keywords:** Immunonutrition, Plasma arginine, Plasma citrulline, Critical care, SOFA score

## Abstract

**Background:**

Restoring plasma arginine levels through enteral administration of L-citrulline in critically ill patients may improve outcomes. We aimed to evaluate whether enteral L-citrulline administration reduced organ dysfunction based on the Sequential Organ Failure Assessment (SOFA) score and affected selected immune parameters in mechanically ventilated medical intensive care unit (ICU) patients.

**Methods:**

A randomized, double-blind, multicenter clinical trial of enteral administration of L-citrulline versus placebo for critically ill adult patients under invasive mechanical ventilation without sepsis or septic shock was conducted in four ICUs in France between September 2016 and February 2019. Patients were randomly assigned to receive enteral L-citrulline (5 g) every 12 h for 5 days or isonitrogenous, isocaloric placebo. The primary outcome was the SOFA score on day 7. Secondary outcomes included SOFA score improvement (defined as a decrease in total SOFA score by 2 points or more between day 1 and day 7), secondary infection acquisition, ICU length of stay, plasma amino acid levels, and immune biomarkers on day 3 and day 7 (HLA-DR expression on monocytes and interleukin-6).

**Results:**

Of 120 randomized patients (mean age, 60 ± 17 years; 44 [36.7%] women; ICU stay 10 days [IQR, 7–16]; incidence of secondary infections 25 patients (20.8%)), 60 were allocated to L-citrulline and 60 were allocated to placebo. Overall, there was no significant difference in organ dysfunction as assessed by the SOFA score on day 7 after enrollment (4 [IQR, 2–6] in the L-citrulline group vs. 4 [IQR, 2–7] in the placebo group; Mann‒Whitney *U* test, *p* = 0.9). Plasma arginine was significantly increased on day 3 in the treatment group, while immune parameters remained unaffected.

**Conclusion:**

Among mechanically ventilated ICU patients without sepsis or septic shock, enteral L-citrulline administration did not result in a significant difference in SOFA score on day 7 compared to placebo.

*Trial registration*: ClinicalTrials.gov Identifier NCT02864017 (date of registration: 11 August 2016).

**Supplementary Information:**

The online version contains supplementary material available at 10.1186/s13054-023-04651-y.

## Introduction

Several observations indicate that a significant proportion of patients admitted to the intensive care unit (ICU) will develop temporary immune deficiencies [1]. The strong impact of critical illness on immune function is responsible for persistent organ dysfunction and a higher risk for ICU-acquired infections, leading researchers to develop an immune-enhancing diet. Among immune-modulating nutrients, L-arginine-enriched formulas have been used to restore the arginine deficiency observed at admission in critically ill patients, which is associated with worse outcomes [2, 3]. In addition to its crucial role in protein synthesis, L-arginine is an amino acid that serves as a precursor for multiple metabolites with strong immunomodulatory properties [4]. Under normal conditions, L-arginine is synthesized de novo and is therefore not diet dependent. However, during the critically ill state, enhanced arginase activity, which converts L-arginine to L-ornithine and urea, is responsible for a significant decrease in L-arginine availability, which becomes a problem since endogenous L-arginine synthesis no longer supplies enough of it [3, 5–7].

Although the results have been confounded by grouping different formulas and different types of patients together, harmful effects have been suspected from the use of L-arginine-containing formulas in critically ill patients, especially in patients with sepsis or septic shock [8–10]. In a randomized, double-blinded monocentric therapeutic trial conducted in 30 medical ICU patients, comparing standard enteral nutrition plus L-arginine to standard enteral nutrition plus placebo, administration of L-arginine significantly increased ornithine synthesis and the plasma concentration of ornithine, suggesting a preferential use by the arginase pathway, while L-arginine plasma levels and immune functions were unaffected [11]. Importantly, based on a sequential organ failure assessment (SOFA) score evaluation, L-arginine administration did not seem clinically deleterious in this selected medical ICU subpopulation. Along these lines, a recent study found that supplementation with L-citrulline, which is converted to L-arginine through the activity of argininosuccinate synthetase and argininosuccinate lyase and does not undergo first-pass hepatic metabolism, was more efficient than L-arginine at increasing plasma L-arginine [12].

We aimed to evaluate whether L-citrulline administration reduced organ dysfunction as defined by the SOFA score and whether it affected selected immune parameters associated with outcomes, such as interleukin (IL)-6 and monocytic expression of HLA-DR, in mechanically ventilated medical ICU patients. Therefore, we conducted an enteral nutrition intervention study comparing standard enteral nutrition enriched with L-citrulline with standard enteral nutrition plus isonitrogenous isocaloric placebo in mechanically ventilated critically ill patients without sepsis or septic shock.

## Methods

### Study design

IMMUNOCITRE was a double-blind randomized placebo-controlled trial involving four intensive care units (ICUs) in France. The study protocol was approved by an independent ethics committee (*Comité de Protection des personnes*, France; reference: 2016–895) and by regulatory authorities (reference: 150806B-42). Signed informed consent was obtained from all the participants or their legally authorized representatives prior to enrollment and randomization. This trial was registered at ClinicalTrials.gov, No. NCT02864017.

Because flow cytometry analysis for immune monitoring required live cell samples, patients were included only from Monday to Wednesday so that each sample (day 1, day 3, and day 7) fell on a regular workday.

### Study population

Patients admitted to the ICU were eligible for the study if they (1) were aged 18 years or older, (2) had a time from symptom onset to enrollment less than five days, (3) required invasive mechanical ventilation with a predicted duration of at least two days [13], and (4) required exclusive enteral nutrition. Patients were not eligible if they (1) had a body mass index > 40 kg/m^2^, (2) were immunocompromised (hematological disorder, autoimmune disease, immunodeficiency, immunosuppressive therapy), (3) had a contraindication to enteral nutrition [14], (4) had undergone surgery within one month prior to enrollment, (5) were pregnant, or (6) were admitted with sepsis or septic shock according to the Sepsis-3 criteria [15]. Patients were also excluded if immunosuppressive therapy, such as chemotherapy, cyclophosphamide, or high-dose corticosteroid therapy (> 0.5 mg/kg/day), was required during the ICU stay.

### Randomization and masking

Patients were randomized in a 1:1 ratio to either L-citrulline (intervention group) or placebo (control group). The randomization sequence was generated using SAS 9.4 software (SAS Institute, Cary, NC, USA) and used blocks of size 6, stratified by site.

To maintain blinding, treatments were prepared by a nurse from another unit who was not involved in the clinical trial.

### Study interventions

The study drug was 5 g of enterally administered L-citrulline (Proteocit, Citrage, Boissy St Léger-France) every 12 h for 5 days, starting within 24 h of inclusion. L-Citrulline was administered enterally through nasogastric tube feeding in 30 s. Patients randomized to placebo received a standard isonitrogenous, isocaloric preparation of nonessential amino acids, including alanine, glycine, aspartic acid, and proline, in a matching volume (50 mL) using the same techniques at the same time points. All patients included in the study received enteral nutrition to achieve recommended caloric and protein intakes [16].

### Interventions

At inclusion (before treatment administration), the organ dysfunction severity was evaluated with the SOFA score together with an assessment of plasma concentrations of selected amino acids (arginine, citrulline, glutamate, glutamine, ornithine, proline, and tryptophan (and kynurenine)), interleukin-6 (IL-6), and monocytic human leukocyte antigen-DR expression (mHLA-DR). The same evaluation was repeated on day 3 (during treatment) and on day 7 (after treatment).

### Outcomes

The primary outcome was the SOFA score on day 7. In cases of death or discharge from the ICU before day 7, the last-observation-carried-forward method was used.

The secondary outcomes were (1) SOFA score on day 3; (2) proportion of patients with an improvement in the SOFA score (defined as a decrease in total SOFA score by 2 points or more between day 1 and day 7 or discharge from the ICU due to improved clinical status); (3) mHLA-DR expression on day 1, day 3, and day 7; (4) plasma concentration of IL-6 on day 1, day 3, and day 7; (5) plasma concentrations of amino acids (arginine, citrulline, glutamate, glutamine, ornithine, proline, and tryptophan (and kynurenine)) on day 1, day 3, and day 7; (6) incidence of ICU-acquired infections; (7) length of ICU or hospital stay; (8) hospital and ICU survival; and (9) length of mechanical ventilation. All ICU-acquired infections were recorded, including ventilator-associated pneumonia, postextubation pneumonia, bloodstream infections, intra-abdominal infection, *Clostridium difficile* infection, upper genitourinary tract infection, skin and soft-tissue infection, and other infections, adapted from the International Sepsis Forum [17].

### Protocole changes

Day 28 mortality was measured, as a post hoc analysis of our data since day 28 mortality was not listed as a prespecified outcome in our trial.

As additional immune parameters, three unspecified exploratory outcomes were added to the trial and were evaluated on day 1, day 3, and day 7: number of circulating monocytic myeloid-derived suppressor cells (M-MDSCs), which was evaluated only in one center (Rennes); total lymphocyte count; and indoleamine-pyrrole 2,3-dioxygenase (IDO) activity.

During the peer review process, changes have been made to the statistical analysis plan. A sensitivity analysis on the primary endpoint has been added: an Analysis of Covariance (ANCOVA) adjusting for the SOFA score at day 1 was used to analyze SOFA score at day 7. For secondary endpoint, a log transformation was applied to deal with non-normal distribution of the following variables: arginine, citrulline, glutamine, ornithine, and kynurenine.

### Immune monitoring and amino acid plasma level measurements

Blood samples were obtained on day 1, day 3, and day 7. Notably, blood samples were collected prior to initiation of treatment on day 1 and immediately prior to treatment administration on day 3.

#### Cytokine quantification

Plasma levels of IL-6 were quantified by ELISA (DuoSET ELISA, R&D System, Abingdon, UK).

#### Amino acid quantification

Plasma concentrations of arginine, citrulline, glutamate, glutamine, ornithine, proline, and tryptophan (and kynurenine) were determined by ion-exchange chromatography. IDO activity was evaluated by the ratio of kynurenine to tryptophan in plasma [18].

#### Gating strategy for the identification of monocytic myeloid-derived suppressor cells (M-MDSCs) and monocyte subsets

Peripheral blood was labeled with CD45, CD14, CD16, CD66b, CD335, and HLA-DR. After exclusion of doublets from the cell gate, granulocytes, T cells and NK (CD66b+CD335+CD14−), and basophils, lymphocytes and granulocytes (CD14-HLA-DR-) were excluded from the Monogate. Monocytes have been classified into three subtypes: classic (CD14+CD16−), intermediate (CD14+CD16+), and nonclassic monocytes (CD14dimCD16+). M-MDSC subsets were defined as CD14+ and HLA-DRlow cells. (Gating strategies are represented in Additional file 1: Figure S1.) Notably, since mHLA-DR expression data were generated by different instruments without standardized flow cytometric protocol between centers, the results are expressed as fold changes.

### Safety analysis

Adverse events and serious adverse events were coded according to the Medical Dictionary for Regulatory Activities (MedDRA) version 22.1 and are presented by system organ class (SOC) and preferred terms (PT).

### Statistical analysis

Following our pilot study [11], the IMMUNOCITRE study was designed to detect a standardized mean difference of 0.66 [i.e., SOFA score difference of 2 points assuming a common standard deviation (SD) of 3]. A sample size of 60 patients per group was needed to detect this difference with 95% power (a two-sided 5% significance level).

Statistical analyses were performed on an intention-to-treat basis. Baseline dichotomous characteristics are described as number (percentage), and quantitative variables are described as mean [standard deviation] or median [interquartile range].

Dichotomous variables were compared with the *χ*^2^ test (or Fisher’s exact test, as appropriate). Continuous variables were compared with the t test (or Wilcoxon rank-sum test in case of nonnormal distribution). For time-to-event variables, Kaplan‒Meier estimates were used, and the groups were compared with a log-rank test. Plasma concentrations of selected amino acids (arginine, citrulline, glutamate, glutamine, ornithine, proline, and tryptophan (and kynurenine)) and IL-6, quantification of mHLA-DR, and lymphocyte and M-MDSC counts were analyzed using mixed-model repeated measures (fixed effect: L-citrulline vs. placebo; repeated measures: day 1, day 3, and day 7).

### Role of the funding source

The funding source—National Clinical Research Hospital Program of the French Ministry of Health—had no role in the design or conduct of the study; collection, management, analysis, or interpretation of the data; preparation, review, or approval of the manuscript; or decision to submit the manuscript for publication. The corresponding author had full access to all the data in the study and takes responsibility for the integrity of the data and the accuracy of the data analysis.

## Results

### Patients

**Fig. 1:**
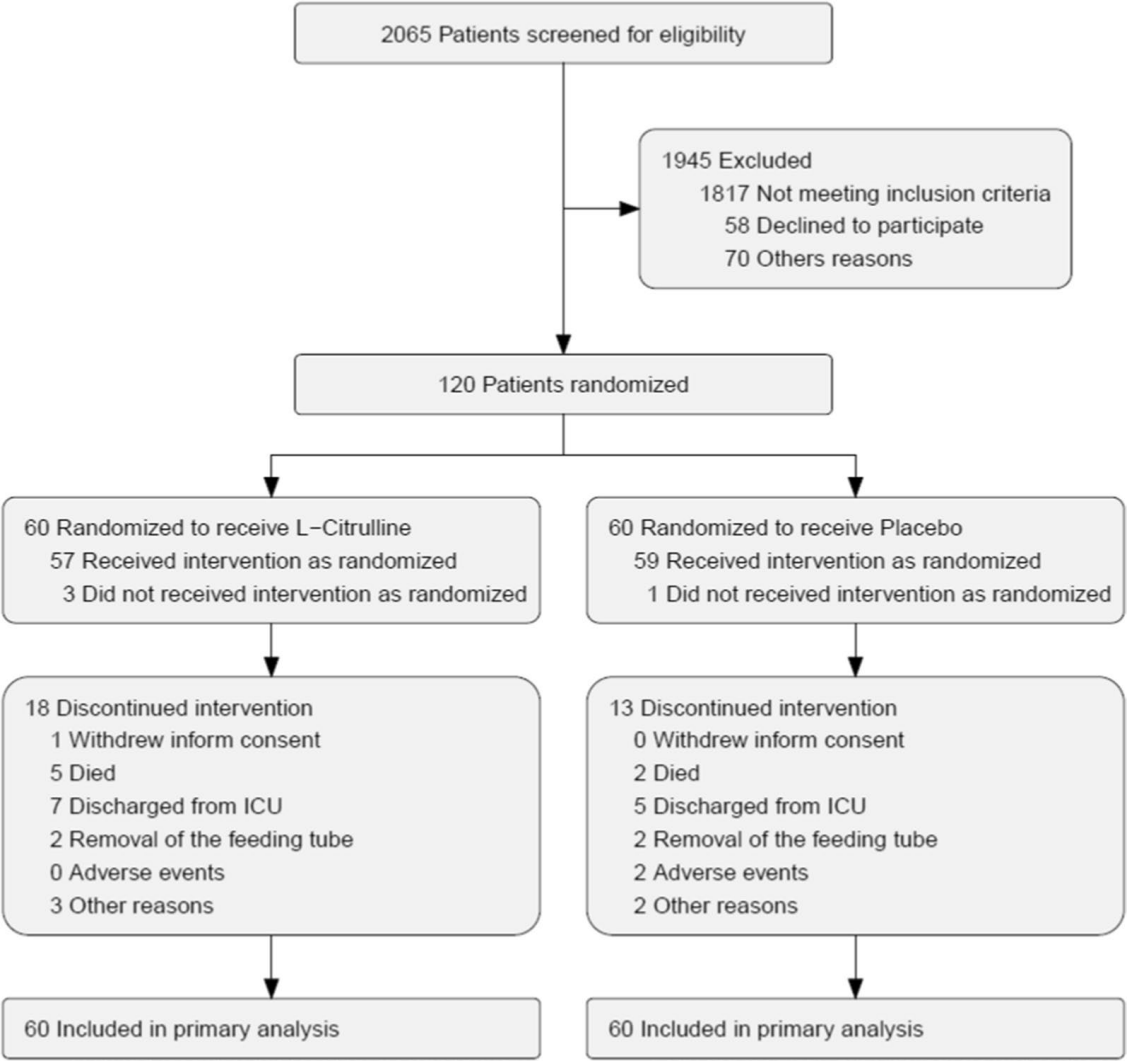
Flowchart of patients in the IMMUNOCITRE trial

From September 2016 to April 2019, 2065 patients from the four centers were screened for eligibility. (Most common screen failures were expected duration of MV < 48 h (26%), sepsis (18%), immunosuppression (15%), and moribund status (12%).) Among these, 120 (6%) fulfilled the enrollment criteria and were randomly assigned to receive enteral administration of L-citrulline (5 g/12 h) or isonitrogenous, isocaloric placebo in matching volumes for 5 days (Fig. 1). The two groups had similar characteristics at baseline (Table 1). After inclusion in the study, four patients did not receive the treatment as allocated for the following reasons: immunosuppression (1 patient), septic shock (2 patients), and intestinal obstruction (1 patient). The median time from invasive mechanical ventilation initiation to inclusion was 1 day [IQR, 0–2] in both groups. Median daily energy (kcal/day) and protein (g/day) intakes during study drug administration are reported in Additional file 4: Table S1 and were comparable between the two groups. The treatment consisted of 10 administrations (2 per day for 5 days). In the placebo group, the median number of administrations was 10 [range: 0–10; IQR: 9–10]. In the L-citrulline group, the median number of administrations was also 10 [range: 0–10; IQR: 7–10].

**Table 1:** Baseline Characteristics in a study of Effect of enteral citrulline administration in critically ill patients under mechanical ventilation vs standard of care nutrition on SOFA score at day 7

	L-citrulline (*n* = 60)	Control (*n* = 60)
*Demographic data, No. (%)*		
Age (y), median (IQR)	62 [48–74]	63 [53–70]
Male	39 (65%)	37 (62%)
Female	21 (35%)	23 (38%)
BMI (kg/m^2^), mean ± SD,	26.3 ± 4.9	27.4 ± 5.4
Reason for ICU admission		
Cardiac arrest	11 (18%)	10 (17%)
Cardiogenic shock	2 (3%)	5 (8%)
Neurologic	18 (30%)	12 (20%)
Respiratory	18 (30%)	20 (33%)
Abdominal	2 (3%)	7 (11%)
Metabolic or acute intoxication	9 (15%)	6 (10%)
SOFA score, median [IQR]	7 [5–9]	8 [6–11]
*Delay between mechanical ventilation initiation and randomization (days),* *median [IQR]*	1 [0–2]	1 [0–2]
*Plasma amino acid concentrations*		
L-citrulline (μmol/l), (missing data) median [IQR]	(13) 14.3 [10.2–21.0]	(11) 17.1 [13.4–26.4]
Arginine (μmol/l), (missing data) median [IQR]	(13) 35.3 [22.6–50.1]	(11) 36.7 [26.2–55.7]
Glutamate (μmol/l), (missing data) median [IQR]	(13) 35.7 [20.9–50.9]	(11) 39.3 [23.0–52.4]
Glutamine (μmol/l), (missing data) median [IQR]	(13) 458.7 [357.3–540.8]	(11) 428.0 [321.6–576.3]
Ornithine (μmol/l), (missing data) median [IQR]	(13) 48.7 [30.3–61.9]	(11) 49.2 [32.9–63.3]
Proline (μmol/l), (missing data) median [IQR]	(13) 107.8 [81.8–150.1]	(11) 116.8 [102.3–143.7]
Tryptophan (μmol/l), (missing data) median [IQR]	(13) 32.5 [24.9–43.6]	(13) 28.9 [20.3–36.0]
Kynurenine (μmol/l), (missing data) median [IQR]	(13) 2.4 [1.4–3.4]	(11) 2.3 [1.5–3.3]

*BMI* body mass index, calculated as weight in kilograms divided by height in meters squared, *IQR* interquartile range, *SOFA* Sequential Organ Failure Assessment

### Primary and secondary outcomes

**Fig. 2:**
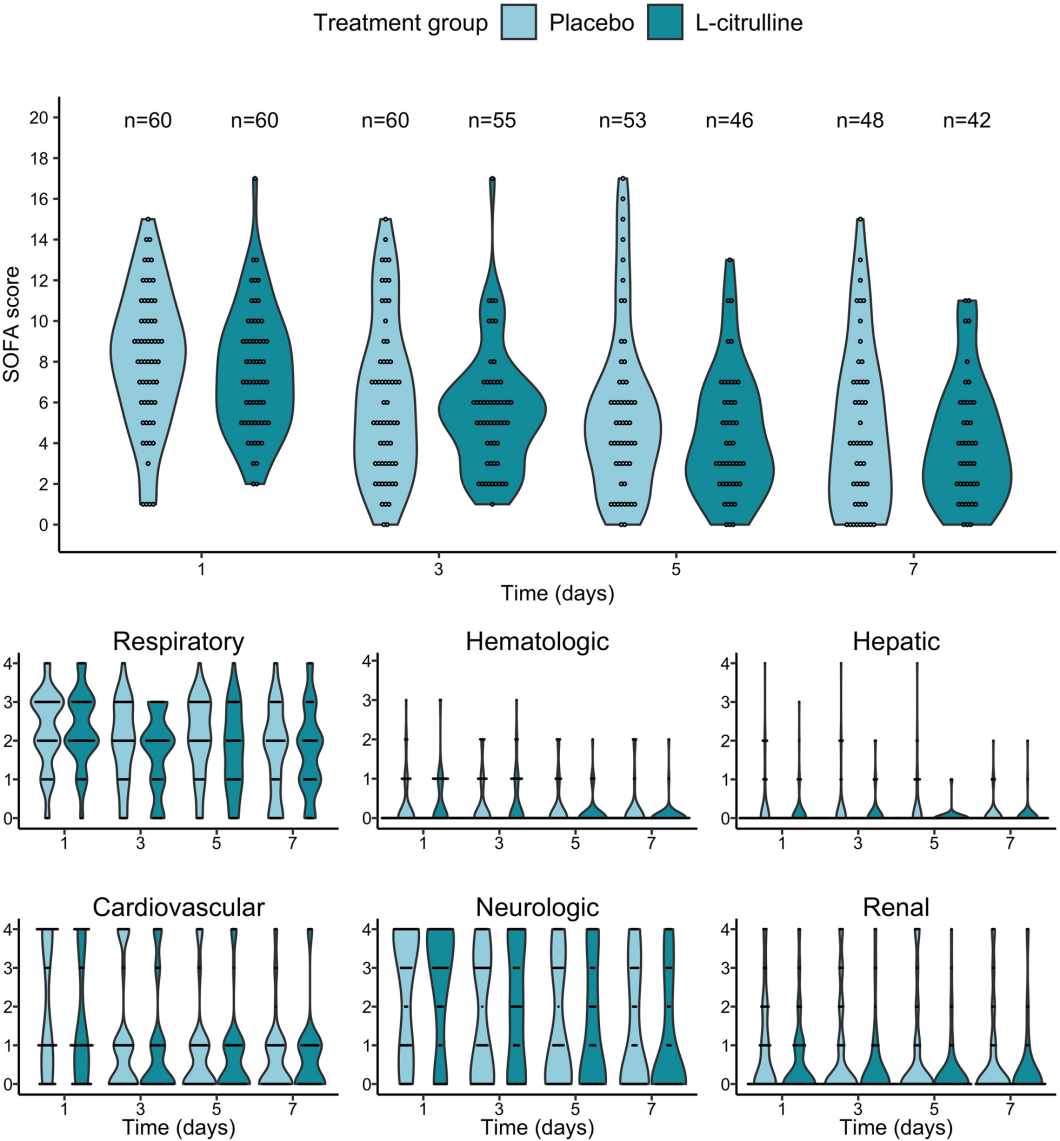
Sequential organ failure (SOFA) score evolution by treatment group from randomization to day 7. The primary outcome was the SOFA score on day 7. Since SOFA score was nonnormally distributed, the Mann‒Whitney *U* test was used to compare it between groups. There was no significant difference in the SOFA score on day 7 between the two groups. Distinct scores for each of the 6 different organ systems (respiratory, hematology, hepatic, cardiovascular, neurological, and renal) are detailed under the full SOFA score

On day 7, the SOFA score did not differ between patients with and without L-citrulline (4 [IQR, 2–6] vs. 4 [IQR, 2–7], respectively-; Mann‒Whitney *U* test, *p* = 0.9) (Fig. 2). A sensitivity analysis by means of analysis of covariance (ANCOVA) with the baseline SOFA score as a covariate confirmed the results of the primary non-parametric analysis (*p* value = 0.701). Likewise, no difference in the SOFA score was observed on day 3, and the proportion of patients with an improvement in the SOFA score was not significantly different between the two groups (Table 2). No significant differences in duration of ventilation, incidence of nosocomial infection during ICU stay or duration of ICU stay were found between the two groups. Hospital mortality was 18% (11/60) in the placebo group versus 22% (13/60) in the L-citrulline group.

**Table 2:** Primary and secondary outcomes in a study of Effect of enteral citrulline administration in critically ill patients under mechanical ventilation vs standard of care nutrition on SOFA score at day 7

	L-citrulline (*n* = 60)	Control (*n* = 60)	*p* value
*Primary outcome*			
SOFA at day 7	4 [2–6]	4 [2–7]	0.77
*Secondary outcomes*			
SOFA at day 3	6 [4–7]	5 [3–8]	0.82
Rate of patients with a decrease of SOFA score ≥ 2 between day 1 and day 7	35 (58.3%)	38 (63.3%)	0.73
Nosocomial infections acquisition	8 (13.3%)	17 (28.3%)	0.18
Pneumonia	8 (13.3%)	10 (16.7%)	0.66
Positive urine culture	0 (0%)	5 (8.3%)	0.06
Any bacteremia	0 (0%)	2 (3.3%)	0.5
Hospital mortality	13 (22%)	11 (18%)	0.46
ICU mortality	12 (20%)	11 (18%)	0.81
Mechanical ventilation duration (days)	7 [3–11]	8 [5–14]	0.08
ICU length of stay to discharge or death, median (IQR)	9 [5–16]	11 [7–18]	0.10
Hospital length of stay to discharge or death, median (IQR)	15 [9–25]	19 [10–28]	0.11

*SOFA* Sequential Organ Failure Assessment, *ICU* Intensive Care Unit

**Fig. 3:**
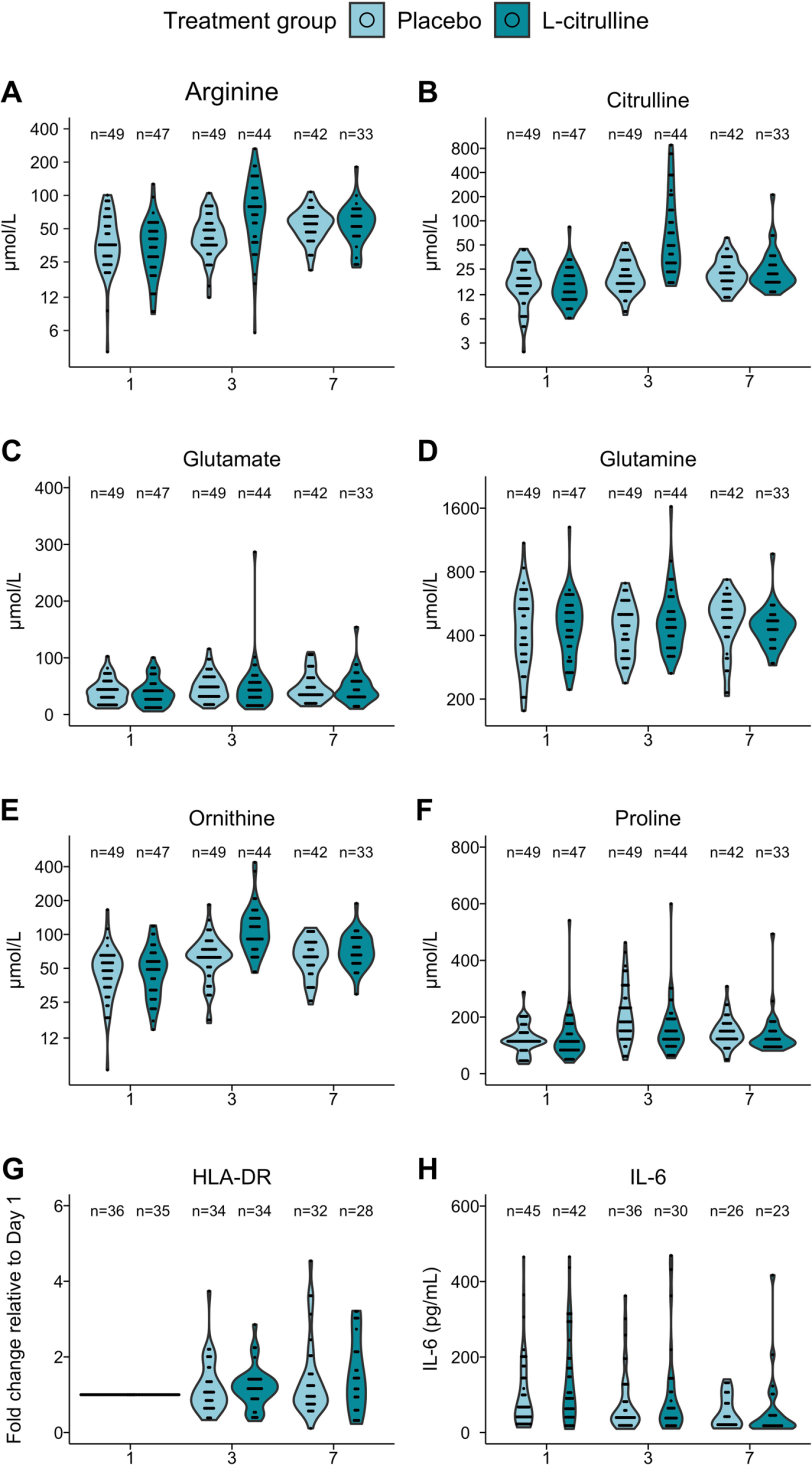
**A–F** Plasma amino acid levels by treatment group from randomization to day 7. **G** Monocytic human leukocyte antigen-DR expression (mHLA-DR) fold change relative to day 1 by treatment group from randomization to day 7. **H** Plasma interleukin-6 (IL-6) by treatment group from randomization to day 7

Analysis of plasma amino acid levels showed that there was no group effect and no time × group interaction for glutamate plasma concentrations. In contrast, for citrulline, proline, and ornithine, there was a group effect and a time × group interaction. For arginine, there was a time × group interaction, plasma arginine being significantly higher on day 3 in the L-citrulline group (42.4 [34.3–54.0] vs. 75.3 [42.5–113.0] μmol/l, p < 0.0001) (Fig. 3, Additional file 4: Table S2).

#### Additional secondary outcomes

There were no differences between the two groups in mHLA-DR expression or IL-6 concentration. Along these lines, no differences were found between the two groups in lymphocyte count or M-MDSC and IDO activity (Additional file 2: Figure S2).

The Kaplan‒Meier curves for overall survival from randomization (day 0) to day 28 are presented in Additional file 3: Figure S3 and showed no difference (HR = 1.24 [95% CI 0.56–2.77]).

### Adverse events

In the L-citrulline group, 38 adverse events occurred in 23 (40%) patients, and in the placebo group, 54 adverse events occurred in 27 (46%) patients. Serious adverse events occurred in 13 (23%) patients in the L-citrulline group and 15 (25%) in the placebo group. None of these adverse events were causally related to the study product. Adverse events that occurred in more than one patient are presented in Additional file 4: Table S3.

## Discussion

In this multicenter randomized clinical trial conducted in medical ICU patients under invasive mechanical ventilation without sepsis or septic shock, enteral administration of L-citrulline for 5 days did not result in a significantly lower SOFA score on day 7 compared with standard care even though it significantly increased plasma L-arginine concentration.

### SOFA score as an endpoint

We selected the SOFA score as the primary and major secondary endpoint for several reasons. First, although initially described as a sepsis-related organ dysfunction measure, the utility of the SOFA score for severity assessment in a wide range of critical illnesses has been recognized and has been effectively used in several clinical trials in nonseptic critically ill patients [17]. Second, the SOFA score was designed to describe a sequence of complications of critical illness, and the score has been used to evaluate the effects of treatment on organ dysfunction. Although a treatment that improves the SOFA score may not necessarily reduce mortality, or vice versa, a recent systematic review indicated that the delta SOFA score, as we used in our study, reliably reflects between-group differences in mortality and describes the change in organ function over time [17, 19, 20]. Notably, we selected as the primary measure the SOFA score on day 7 and not the difference between the SOFA score on day 1 and the SOFA score on day 7 because IMMUNOCITRE was a randomized clinical trial, so no difference in the SOFA score between the 2 groups was expected at baseline. Even so, we explored the change in the SOFA score between day 1 and day 7 as a secondary endpoint to determine the rate of improvement in the ICU (defined as a decrease in total SOFA score by 2 points or more between day 1 and day 7).

### L-citrulline and L-arginine in critically ill patients

We believe that L-citrulline administration along with a significant increase in plasma L-arginine concentrations is not harmful to medical ICU patients the way it is in other patients [21]. This is an important result since immune-enhanced diets, especially those enriched in L-arginine, have been deemed responsible for increased mortality in critically ill patients [22]. In an interim analysis of a randomized multicenter clinical trial comparing the mortality of critically ill patients given either enteral feeding with an immune-enhancing formula (mainly enriched in L-arginine) or standard parenteral nutrition, Bertolini et al. found that the immune-enhancing formula was associated with increased mortality of patients admitted to the ICU for septic shock [9, 10]. In addition, in three different large clinical trials, concerns were raised about an association between immune-enhancing diets and the worst outcome in ICU patients [23–25]. For these reasons, although none of the trials measured L-arginine plasma levels and therefore failed to demonstrate that L-arginine was effectively responsible for the increased mortality, immune-enhancing nutrition is not recommended in patients with sepsis. Consequently, we decided not to include patients admitted for sepsis or septic shock.

Importantly, L-citrulline administration significantly increased plasma levels of L-arginine, suggesting it could be an interesting therapeutic in critical conditions associated with L-arginine deficiency, which itself has been associated with mortality [3, 26]. Under normal conditions, L-arginine is synthesized de novo and therefore does not need to come from the diet [3, 6]. In critical illness, enhanced arginase activity, which converts L-arginine to L-ornithine and urea, is responsible for a significant decrease in L-arginine availability, making this amino acid essential once the endogenous L-arginine synthesis capacity is exceeded [11, 27]. Of note, since L-citrulline is not only the precursor of L-arginine but also its metabolic product, overuse of L-arginine through the arginase pathway could be responsible for a decreased availability of L-arginine for L-citrulline synthesis and could therefore be responsible for low L-citrulline plasma levels, as we found at baseline in our study and others have reported [28–30]. Recently, several studies have found that supplementation with L-citrulline, which is converted to L-arginine through the activity of argininosuccinate synthase and argininosuccinate lyase and does not undergo hepatic first-pass metabolism, was more efficient than L-arginine at increasing plasma L-arginine [31]. Along these lines, using a mouse model of sepsis, we recently found that L-citrulline enteral administration increased plasma concentrations of L-arginine and enhanced immune function [32, 33]. This is noteworthy because enteral L-arginine administration has failed to increase arginine plasma levels in critically ill patients, mainly due to enhanced arginase 1 activity along with first-pass hepatic metabolism [11, 34]. However, a rapid clearance of administered amino acids and their metabolites (i.e., 3 h or less) along with administration of L-citrulline for 5 days could have been responsible for the lack of difference observed in plasma L-arginine on day 7 between the two groups [35].

### Assessment of immune dysfunction in critically ill patients

In the present study, the potential immune effects of L-citrulline administration and higher plasma L-arginine were explored by measuring validated biological markers. IL-6 is considered a reliable marker of illness severity, and monocytes express HLA-DR molecules, which are responsible for antigen presentation to T cells and are a validated marker of immune function associated with the risk of nosocomial infection acquisition and mortality in critically ill patients [36, 37]. Both markers presented a similar evolution in the L-citrulline and placebo groups within the first week in the ICU and returned to normal along with SOFA improvement. Three more immunological parameters were selected as exploratory outcomes. First, since L-arginine availability can modulate T-cell function, we measured the number of circulating lymphocytes. For instance, human T cells stimulated and cultured in the absence of L-arginine lose the expression of the TCR ζ-chain (CD3ζ) and have impaired proliferation and decreased cytokine production [38]. We hypothesize that, although significant, the increase in the plasma level of arginine was moderate and might have been too transient to induce significant changes in both the lymphocyte ability to proliferate and the lymphocyte apoptosis rate. Second, we observed an expansion of M-MDSCs, a robust marker of acquired immune dysfunction in critically ill patients that has been associated with mortality and secondary infection acquisition [39]. Interestingly, we found that the expansion of M-MDSCs, which could be initiated by L-arginine deprivation [27], occurred several days after admission. This is noteworthy because upregulation of arginase 1 in MDSCs or the release of arginase 1 from the destruction of erythrocytes or hepatocytes induces L-arginine depletion [40]. Finally, we measured indoleamine 2,3 dioxygenase (IDO) activity by calculating the kynurenine/tryptophan ratio. The expression of IDO activity by several immune cells, such as monocytes or MDSCs, has strong immunomodulatory effects on T cells related to the degradation of the essential amino acid tryptophan [18]. Notably, arginine metabolism affects IDO activity [41], which has been associated with mortality in critically ill patients [18, 42]. In our study, none of these three immunological parameters were affected by L-citrulline administration, which argues for not administering it in critically ill nonseptic patients. These results are in line with other clinical trials that failed to demonstrate any biological effects of immune-enhancing diets in critically ill patients [8, 24, 43].

### Strengths of the study

One of the strengths of our study is the design of the trial. In the medical ICU, the time between disease deterioration and admission to the ICU may vary widely. Therefore, the metabolic status of patients may differ greatly from one study to another and may be responsible for introducing heterogeneity within the study. To avoid this shortcoming, we selected only patients who had a time from symptom onset to enrollment less than five days, allowing us to select only patients who were not at risk of hospital-acquired denaturation and immune dysfunction before study enrollment [44, 45]. Furthermore, although we acknowledge that immunocompromised patients represent a growing proportion of critically ill patients with a high risk of secondary infection acquisition, we decided not to include these specific patients due to the particular metabolic profile associated with acquired immune dysfunction.

### Limitations of the study

First, the number of screen failure is high and may cast doubt on the general applicability of our results. First, we estimated the SOFA score at admission based on two previous studies performed in medical ICU patients [11, 46]. Unfortunately, the median SOFA score at admission was lower than expected, although it was accurately estimated on day 7. This SOFA overestimation could be partly explained by the noninclusion of patients with septic shock, who have higher SOFA scores at admission than critically ill nonseptic patients. Notably, a significant proportion of patients admitted for cardiac arrest and neurologic reasons with irrecoverable brain injury at admission were included in our study. This could have been responsible for the lack of improvement in the SOFA score neurological assessment, which may have significantly influenced the SOFA scores we saw on day 7. We only administered L-citrulline for 5 days, and a longer duration of treatment should be considered. We chose this time based on previous studies [3, 11, 12, 46]. We hypothesized that restoring the plasma level of arginine through L-citrulline administration would improve SOFA score parameters within 24 h after the last administration. Since the SOFA score is calculated based on the most severe value of each subscore in the 24 h preceding the day of calculation, we selected day 7 as the primary endpoint. In addition, the actual protein intake may have been too low for L-citrulline to exert its anabolic effects [21].

## Conclusions

Among mechanically ventilated adult medical ICU patients without sepsis or septic shock, enteral citrulline administration did not result in a statistically significant reduction in SOFA score during the first 7 days after enrollment or a significant improvement in selected immunological parameters. Our data suggest no beneficial effect of L-citrulline on the outcome of critically ill patients treated with invasive mechanical ventilation.

### Supplementary Information

**Additional file 1: Figure S1**. Gating strategy for the identification of monocyte subsets and monocytic myeloid-derived suppressor cells (M-MDSCs). Peripheral blood was labeled with CD45, CD14, CD16, CD66b, CD335, and HLA-DR. After exclusion of doublets into the cell gate, granulocytes, T cells and NK (CD66b+CD335+CD14−), and basophils, lymphocytes and granulocytes (CD14-HLA-DR-) were excluded from the Monogate. Monocytes have been classified into three subtypes: classic (CD14+CD16−), intermediate (CD14+CD16+), and nonclassic monocytes (CD14dimCD16+). M-MDSC subsets have been defined as CD14+ and HLA-DRlow cells..

**Additional file 2: Figure S2**. Secondary outcome evolution from randomization to day 7 in the enteral citrulline supplementation group versus the placebo group of mechanically ventilated critically ill patients in the IMMUNOCITRE Randomized Clinical Trial. **A.** Total lymphocyte count. **B.** Circulating monocytic myeloid-derived suppressor cell (M-MDSC) count. **C.** Indoleamine-pyrrole 2,3-dioxygenase (IDO) activity (evaluated by the ratio between plasma concentrations of kynurenine and tryptophan).

**Additional file 3:: Figure S3**. Kaplan‒Meier curves for time to death from randomization (day 0) to day 28 in the enteral citrulline supplementation group versus the placebo group of mechanically ventilated critically ill patients in the IMMUNOCITRE Randomized Clinical Trial.

**Additional file 4**. **eTable 1:** Median [IQR] daily energy (kcal/day) and protein (g/day) intake during study drug administration. **eTable 2.** Plasma concentrations of selected amino acids. **eTable 3.** Adverse Event Definitions.

## Data Availability

The data that support the findings of this study are available from the corresponding author (Prof Jean-Marc Tadié) upon reasonable request.
