# Supplementary figures and images for: Enteral citrulline supplementation versus placebo on SOFA score on day 7 in mechanically ventilated critically ill patients: the IMMUNOCITRE randomized clinical trial
Source: Crit Care. 2023 Oct 3;27:381. doi: 10.1186/s13054-023-04651-y (PMC10546668; doi:10.1186/s13054-023-04651-y)

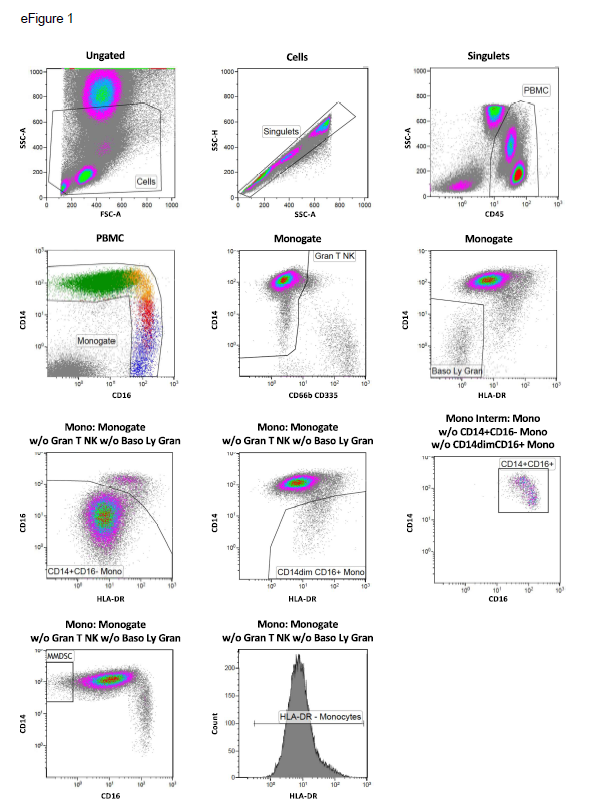

Supplement: Supplementary file 1 — Additional file 1: Figure S1. Gating strategy for the identification of monocyte subsets and monocytic myeloid-derived suppressor cells (M-MDSCs). Peripheral blood was labeled with CD45, CD14, CD16, CD66b, CD335, and HLA-DR. After exclusion of doublets into the cell gate, granulocytes, T cells and NK (CD66b+CD335+CD14−), and basophils, lymphocytes and granulocytes (CD14-HLA-DR-) were excluded from the Monogate. Monocytes have been classified into three subtypes: classic (CD14+CD16−), intermediate (CD14+CD16+), and nonclassic monocytes (CD14dimCD16+). M-MDSC subsets have been defined as CD14+ and HLA-DRlow cells.. [file 13054_2023_4651_MOESM1_ESM.png]

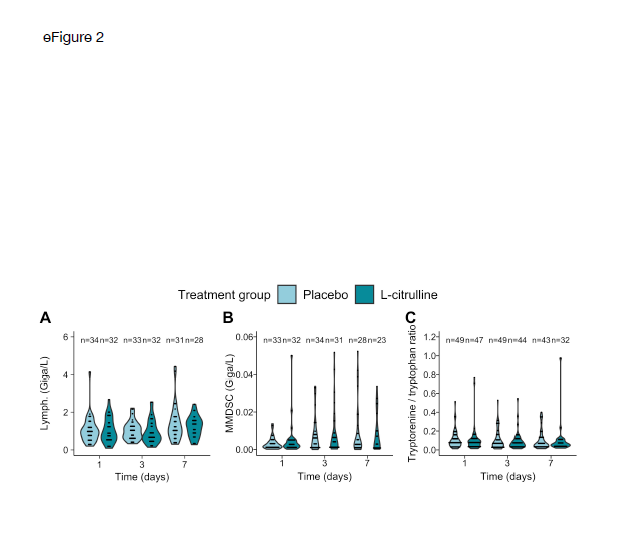

Supplement: Supplementary file 2 — Additional file 2: Figure S2. Secondary outcome evolution from randomization to day 7 in the enteral citrulline supplementation group versus the placebo group of mechanically ventilated critically ill patients in the IMMUNOCITRE Randomized Clinical Trial. A. Total lymphocyte count. B. Circulating monocytic myeloid-derived suppressor cell (M-MDSC) count. C. Indoleamine-pyrrole 2,3-dioxygenase (IDO) activity (evaluated by the ratio between plasma concentrations of kynurenine and tryptophan). [file 13054_2023_4651_MOESM2_ESM.png]

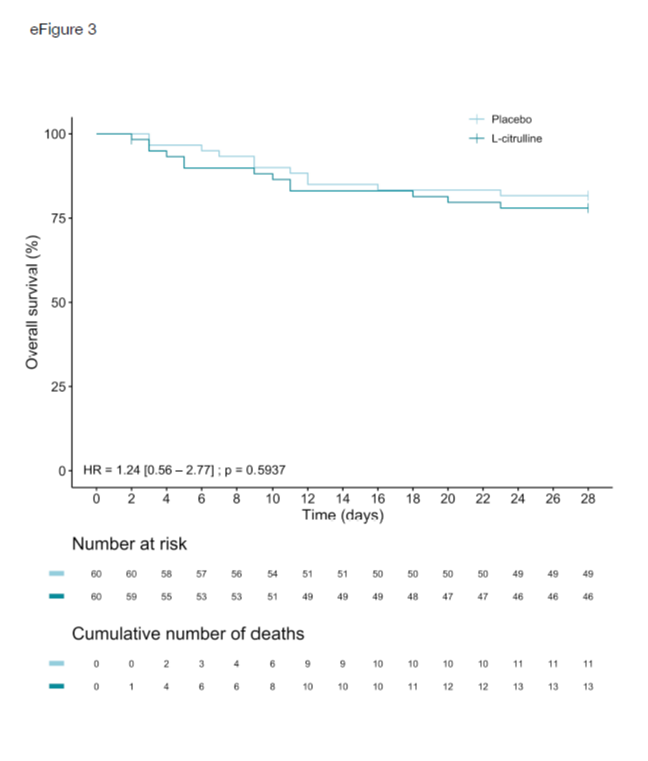

Supplement: Supplementary file 3 — Additional file 3:: Figure S3. Kaplan‒Meier curves for time to death from randomization (day 0) to day 28 in the enteral citrulline supplementation group versus the placebo group of mechanically ventilated critically ill patients in the IMMUNOCITRE Randomized Clinical Trial. [file 13054_2023_4651_MOESM3_ESM.png]
