# Supplementary material for: Enteral citrulline supplementation versus placebo on SOFA score on day 7 in mechanically ventilated critically ill patients: the IMMUNOCITRE randomized clinical trial
Source: Crit Care. 2023 Oct 3;27:381. doi: 10.1186/s13054-023-04651-y (PMC10546668; doi:10.1186/s13054-023-04651-y)
Supplement: Supplementary file 4 — Additional file 4. eTable 1: Median [IQR] daily energy (kcal/day) and protein (g/day) intake during study drug administration. eTable 2. Plasma concentrations of selected amino acids. eTable 3. Adverse Event Definitions. [file 13054_2023_4651_MOESM4_ESM.docx]

**eTable 1: Median [IQR] daily energy (kcal/day) and protein (g/day) intake during study drug administration in a study of Effect of enteral citrulline administration in critically ill patients under mechanical ventilation vs standard of care nutrition on SOFA score at day 7.**

|  |  | L-citrulline (N = 60) | Control (N = 60) |
| --- | --- | --- | --- |
| Day 1 | Energy (kcal/kg) | N = 58  18 [11 – 21] | N = 60  16 [12 – 21] |
|  | Protein (g/kg) | N = 58  0.85 [0.49 – 0.99] | N = 60  0.76 [0.54 – 0.97] |
| Day 2 | Energy (kcal/kg) | N = 55  17 [7 – 23] | N = 58  16 [11 – 21] |
|  | Protein (g/kg) | N = 55  0.78 [0.34 – 1.07] | N = 58  0.77 [0.53 – 0.99] |
| Day 3 | Energy (kcal/kg) | N = 50  17 [8 – 23] | N = 53  17 [12 – 22] |
|  | Protein (g/kg) | N = 50  0.81 [0.38 – 1.07] | N = 53  0.77 [0.55 – 1.02] |
| Day 4 | Energy (kcal/kg) | N = 46  17 [0 – 24] | N = 50  17 [0 – 20] |
|  | Protein (g/kg) | N = 46  0.80 [0.00 – 1.10] | N = 50  0.79 [0.00 – 1.03] |
| Day 5 | Energy (kcal/kg) | N = 45  17 [5 – 23] | N = 48  17 [0 – 22] |
|  | Protein (g/kg) | N = 45  0.77 [0.22 – 1.06] | N = 48  0.77 [0.00 – 1.03] |
| Day 6 | Energy (kcal/kg) | N = 41  17 [0 – 24] | N = 47  16 [0 – 23] |
|  | Protein (g/kg) | N = 41  0.80 [0.00 – 1.10] | N = 47  0.74 [0.00 – 1.09] |

**eTable2.** Plasma concentrations of selected amino acids in a study of Effect of enteral citrulline administration in critically ill patients under mechanical ventilation vs standard of care nutrition on SOFA score at day 7.

|  | L-citrulline | | | Placebo | | | Time | Interaction time*group | Group |
| --- | --- | --- | --- | --- | --- | --- | --- | --- | --- |
|  | Day 1 | Day 3 | Day 7 | Day 1 | Day 3 | Day 7 |  |  |  |
| Arginine (μmol/L) | 35.3  [21.7 – 50.1] | 75.3  [42.5 – 113.0] | 54.9  [42.9 – 72.1] | 36.7  [26.2 – 55.7] | 42.4  [34.3 – 54.0] | 56.5  [44.0 – 65.3] | p < 0.0001 | p < 0.0001 | p = 0.1321 |
| L-Citrulline (μmol/L) | 14.3  [10.2 – 21.0] | 47.5  [27.4 – 136.4] | 20.5  [16.9 – 28.0] | 17.1  [13.4 – 26.4] | 18.7  [15.0 – 26.0] | 21.0  [16.1 – 27.6] | p < 0.0001 | p < 0.0001 | p < 0.0001 |
| Glutamate (μmol/L) | 35.7  [20.9 – 50.9] | 39.5  [23.1 – 57.7] | 37.8  [31.8 – 57.7] | 39.3  [23.0 – 52.4] | 47.6 [30.2 – 61.6] | 39.5  [31.2 – 59.7] | p = 0.0129 | p = 0.8589 | p = 0.8955 |
| Glutamine (μmol/L) | 458.7  [352.3 – 540.8] | 446.8  [353.2 – 528.5] | 440.3  [387.3 – 487.9] | 428.0  [321.6 – 576.3] | 439.3  [342.0 – 512.6] | 499.9  [420.9 – 568.1] | p = 0.1315 | p = 0.1107 | p = 0.4073 |
| Ornithine (μmol/L) | 48.7  [33.07 – 61.9] | 103.1  [81.8 – 142.1] | 70.3  [57.7 – 95.2] | 49.2  [32.9 – 63.3] | 63.8  [53.2 – 77.8] | 66.6  [49.8 – 84.8] | p < 0.0001 | p < 0.0001 | p = 0.0013 |
| Proline (μmol/L) | 107.8  [81.8 – 150.1] | 131.9  [108.5 – 185.7] | 116.8  [103.3 – 150.6] | 116.8  [102.3 – 143.7] | 181.6  [139.6 – 255.8] | 138.2  [113.5 – 171.0] | p < 0.0001 | p = 0.0283 | p = 0.2144 |
| HLA-DR (Fold changes) | 1.0  [1.0 – 1.0] | 1.2  [0.8 – 1.4] | 1.4  [0.9 – 2.1] | 1.0  [1.0 – 1.0] | 1.0  [0.7 – 1.7] | 1.1  [0.8 – 1.6] | p = 0.0336 | p = 0.8677 | p = 0.9320 |
| IL-6 (pg/mL) | 86.3  [45.4 – 188.6] | 50.2  [32.6 – 109.0] | 24.9  [14.6 – 53.4] | 66.0  [37.1 – 145.2] | 42.4  [29.8 – 103.4] | 34.0  [17.1 – 78.9] | p < 0.0001 | p = 0.7288 | p = 0.4268 |
| Kynurenine (μmol/L) | 2.4  [1.4 – 3.4] | 2.2  [1.7 – 3.2] | 2.2  [1.5 – 3.5] | 2.3  [1.5 – 3.3] | 2.2  [1.6 – 4.0] | 2.2  [1.7 – 3.2] | p = 0.7753 | p = 0.9883 | p = 0.7303 |
| Tryptophan (μmol/L) | 32.5  [25.0 – 43.6] | 39.5  [27.0 – 46.8] | 37.8  [31.0 – 43.5] | 28.9  [20.3 – 36.0] | 32.0  [26.2 – 39.8] | 37.5  [27.5 – 44.3] | p = 0.0064 | p = 0.9749 | p = 0.0799 |

**eTable 3**. Adverse Event Definitions in a study of Effect of enteral citrulline administration in critically ill patients under mechanical ventilation vs standard of care nutrition on SOFA score at day 7.

|  | Total | | Placebo | | L-citrulline | | Total | | Placebo | | L-citrulline | |  |
| --- | --- | --- | --- | --- | --- | --- | --- | --- | --- | --- | --- | --- | --- |
| **SOC/PT** | Patients No. (%) | AE No. | Patients No. (%) | AE No. | Patients No. (%) | AE No. | Patients No. (%) | SAE No. | Patients No. (%) | SAE No. | Patients No. (%) | SAE No. | |
|  | 50 (43.1%) | 92 | 27 (45.8%) | 54 | 23 (40.4%) | 38 | 28 (24.1%) | 40 | 15 (25.4%) | 22 | 13 (22.8%) | 18 | |
| Surgical and medical procedures | 5 (4.31%) | 5 | 3 (5.08%) | 3 | 2 (3.51%) | 2 | 5 (4.31%) | 5 | 3 (5.08%) | 3 | 2 (3.51%) | 2 | |
| Therapy cessation | 5 (4.31%) | 5 | 3 (5.08%) | 3 | 2 (3.51%) | 2 | 5 (4.31%) | 5 | 3 (5.08%) | 3 | 2 (3.51%) | 2 | |
| Cardiac disorders | 9 (7.76%) | 12 | 7 (11.86%) | 10 | 2 (3.51%) | 2 | 5 (4.31%) | 7 | 4 (6.78%) | 6 | 1 (1.75%) | 1 | |
| Bradycardia | 2 (1.72%) | 2 | 1 (1.69%) | 1 | 1 (1.75%) | 1 |  |  |  |  |  |  | |
| Cardio-respiratory arrest | 3 (2.59%) | 3 | 3 (5.08%) | 3 |  |  | 3 (2.59%) | 3 | 3 (5.08%) | 3 |  |  | |
| Renal and urinary disorders | 3 (2.59%) | 4 | 3 (5.08%) | 4 |  |  | 1 (0.86%) | 1 | 1 (1.69%) | 1 |  |  | |
| Nervous system disorders | 18 (15.5%) | 19 | 11 (18.6%) | 11 | 7 (12.28%) | 8 | 14 (12.1%) | 15 | 8 (13.56%) | 8 | 6 (10.53%) | 7 | |
| Hypoxic-ischaemic encephalopathy | 7 (6.03%) | 7 | 4 (6.78%) | 4 | 3 (5.26%) | 3 | 7 (6.03%) | 7 | 4 (6.78%) | 4 | 3 (5.26%) | 3 | |
| Ischaemic stroke | 2 (1.72%) | 2 | 1 (1.69%) | 1 | 1 (1.75%) | 1 | 2 (1.72%) | 2 | 1 (1.69%) | 1 | 1 (1.75%) | 1 | |
| Gastrointestinal disorders | 21 (18.1%) | 25 | 12 (20.3%) | 14 | 9 (15.79%) | 11 | 2 (1.72%) | 2 | 1 (1.69%) | 1 | 1 (1.75%) | 1 | |
| Dysphagia | 3 (2.59%) | 3 | 2 (3.39%) | 2 | 1 (1.75%) | 1 |  |  |  |  |  |  | |
| Vomiting | 9 (7.76%) | 10 | 5 (8.47%) | 6 | 4 (7.01%) | 4 |  |  |  |  |  |  | |
| Diarrhoea | 2 (1.72%) | 2 | 2 (3.39%) | 2 |  |  |  |  |  |  |  |  | |
| Small intestine ulcer | 2 (1.72%) | 2 | 1 (1.69%) | 1 | 1 (1.75%) | 1 | 1 (0.86%) | 1 | 1 (1.69%) | 1 |  |  | |
| Hepatobiliary disorders | 2 (1.72%) | 4 |  |  | 2 (3.51%) | 4 |  |  |  |  |  |  | |
| Respiratory, thoracic and mediastinal disorders | 6 (5.17%) | 8 | 1 (1.69%) | 3 | 5 (8.77%) | 5 | 3 (2.59%) | 3 |  |  | 3 (5.26%) | 3 | |
| Acute respiratory distress syndrome | 2 (1.72%) | 2 |  |  | 2 (3.51%) | 2 | 2 (1.72%) | 2 |  |  | 2 (3.51%) | 2 | |
| Vascular disorders | 2 (1.72%) | 2 | 1 (1.69%) | 1 | 1 (1.75%) | 1 | 1 (0.86%) | 1 |  |  | 1 (1.75%) | 1 | |
| Injury, poisoning and procedural complications | 2 (1.72%) | 2 | 2 (3.39%) | 2 |  |  |  |  |  |  |  |  | |
| General disorders and administration site conditions | 5 (4.31%) | 5 | 2 (3.39%) | 2 | 3 (5.26%) | 3 | 4 (3.45%) | 4 | 1 (1.69%) | 1 | 3 (5.26%) | 3 | |
| Multi organ failure | 3 (2.59%) | 3 | 1 (1.69%) | 1 | 2 (3.51%) | 2 | 3 (2.59%) | 3 | 1 (1.69%) | 1 | 2 (3.51%) | 2 | |

* AE: adverse event; SAE: serious adverse event
